# Iron Deficiency Anaemia among Pre-School Children with Sickle Cell Anaemia: Still a Rare Diagnosis?

**DOI:** 10.4084/MJHID.2013.069

**Published:** 2013-11-07

**Authors:** Samuel Olufemi Akodu, Omolara Adeolu Kehinde, Ijeoma Nnenna Diaku-Akinwumi, Olisamedua Fidelis Njokanma

**Affiliations:** Department of Paediatric, Lagos State University Teaching Hospital, Ikeja

## Abstract

**Background:**

The frequent need for blood transfusion in children with SCA creates the impression that IDA is rare in this class of children.

**Objectives:**

The objective of the study is to determine the prevalence of IDA in a population of under-five children with SCA in Lagos, Nigeria.

**Methodology:**

Serum iron, total iron binding capacity, transferrin saturation and serum ferritin were assayed in 97 under-five children with SCAand 97 age/sex matched controls.

The diagnosis of IDA was established based on the following criteria: haemoglobin <11.0 g/dl plus two or more of the following: MCV <70fl, transferrin saturation (Ts) <16% or serum ferritin (SF) <25ng/dL

**Results:**

Overall prevalence of IDA was significantly higher among AA controls. In the younger age group, the prevalence of IDA was significantly higher among HbAA controls while in the older age group the odds of having IDA was three times higher among HbSS subjects but the difference was not statistically significant. Two of the three SCA children with IDA have history of previous blood transfusion.

**Conclusion:**

IDA is uncommon in pre-school aged children with SCA. A multi-centre study is necessary to yield large number of transfused subjects to examine the effects of blood transfusion on prevalence of IDA.

## Introduction

Sickle cell anaemia (SCA) contributes significantly to morbidity and mortality among children in sub-Saharan Africa. Much is known about the disease presentation and end organ manifestation but the iron status in patients with sickle cell disease (SCD) is still a matter of controversy.^1^

Iron status in patients with SCD is still a matter of continuing investigation. While some workers have reported prevalent iron deficiency in sickle cell disease patients, 2 – 5 others have emphasized its rarity.^6^, ^7^

In SCA, chronic haemolysis results in increased availability of iron directly from lysed red cells and also from increased absorption of iron from the gastrointestinal tract.^8^ Additionally, the high load of iron provided by multiple blood transfusions^9^, ^10^ would suggest that iron deficiency is unlikely in SCA. However, in some parts of the world, the frequency of blood transfusion among children with SCA is now less as a result of improved management in recent years.^11^ Reduced frequency of transfusion implies a reduction in sources of iron and therefore, increased vulnerability to iron deficiency anaemia (IDA). This assertion is buttressed by a study in the USA which suggested that iron deficiency was commoner than expected in untransfused children with SCA.^2^ In addition, frequency and need for blood transfusion are not uniform for all children with SCA.

Iron deficiency is a function of the imbalance of iron intake, iron absorption and iron loss.^12^, ^13^ Iron metabolism is unusual in that it is controlled by absorption rather than excretion. 12, 13 In practice iron supplementation is not usually offered in children with SCA for fear of iron overload resulting from an added effect of multiple blood transfusions.

In the current report, anaemia was defined according to the WHO standard of haemoglobin concentration below 11G/dl.^14^ The reduction of body iron has three main stages: (i) iron depletion^15^ which refers to a decrease of iron stores, measured by a reduction in serum ferritin concentration; (ii) iron deficiency,^16^ when storage iron is depleted and there is insufficient iron absorption to counteract normal body losses (at this time, haemoglobin synthesis starts to become impaired and haemoglobin concentrations fall), measured by a reduction in serum ferritin, mean corpuscular volume (MCV) and mean corpuscular haemoglobin (MCH); and (iii) iron deficiency anaemia,^17^ the most severe degree of iron deficiency, which refers to a decrease of iron in the red blood cells, measured by a reduction in serum ferritin, MCV, MCH and haemoglobin level. The distinction between “iron deficiency” and “iron deficiency anaemia” is important. They often go hand in hand, but it is possible to be iron deficient without being anaemic.

Literature suggests that a low MCV for age, transferrin saturation less than 16% and serum ferritin less than 25 ng/ml are each 100% sensitive for IDA in SCD.^2^ – ^5^ With respect to specificity, serum ferritin less than 25 ng/ml is 100% specific for IDA in SCD. In the general children population, a serum ferritin value below 12ng/ml is usually diagnostic of iron deficiency.^2^ However, study has shown that in children with conditions characterized by chronic inflammatory or haemolytic anaemia such as sickle cell disease, a serum ferritin value less than 25ng/ml has been used to predict absent bone marrow iron stores and response to iron therapy.^2^ The transferrin saturation less than 16% and low MCV for age have specificities of 77 – 87% and 97% respectively for IDA in SCD.^2^ – ^5^ The sensitivities of same parameters except serum ferritin among children with haemoglobin AA were not different from that for children with SCD,^18^ In children with haemoglobin genotype AA, a serum ferritin value below 12 ng/ml is 100% sensitive for IDA.^5^ It has been suggested that the use of combination of tests to define iron status in a population improves the precision in diagnosis of IDA.^5^

The identification of IDA in children with SCA is important, as IDA contributes to worsening of inherent anaemia^2^ and may have negative long-term consequences on neurocognitive development^19^, ^20^ and growth.^21^ Studies of iron deficiency among SCA are few in Nigeria.^27^ The purpose of this study is to determine the prevalence of IDA in pre-school aged children with SCA. It is expected that the data generated will provide an assessment tool for the monitoring and assessment of IDA among children with SCA in our area of practice.

## Material and Methods

The cross-sectional study was conducted between December 2009 and February 2010 among children with SCA attending the Sickle Cell Disease Clinic and other Consultant Outpatient Clinics of the Department of Paediatrics, Lagos State University Teaching Hospital (LASUTH), Ikeja in Southwest Nigeria. LASUTH is an urban tertiary health centre. It is a major referral centre serving the whole of Lagos State, which is a major point of entry into Nigeria from different parts of the world and the economic nerve centre of Nigeria.

LASUTH’s Department of Paediatric Sickle Cell Disease Clinic runs every Thursdays of the week. The Sickle Cell Disease clinic caters for children up to age 15 years. On average, each Sickle Cell Disease patients are seen in the sickle cell clinic 4 – 6 times annually. As well as medical check-up packed cell volume estimation are also carried out at each clinic visit. Also routine prophylaxis of antimalarials (daily proguanil 100 – 200mg) in addition to folate (5mg) is being currently offered to children with sickle cell anaemia accessing LASUTH’s Department of Paediatric Sickle Cell Disease Clinic. Those with acute medical problems are managed in the paediatric wards or the Children Emergency Room.

Approval for the study was obtained from the Ethics Committee of LASUTH. Consecutive SCA patients who came for routine follow up clinic and have satisfied the study criteria were recruited. Healthy controls were children with haemoglobin genotype “AA,” from the General Outpatient and follow-up clinics and healthy children attending other specialist clinics (e.g. Paediatric Dermatology Clinic) and were matched for age, and sex. The studied sample size consisted of 194 children, 97 each with haemoglobin genotype SS and AA. Data were summarized for two age categories: age less than two and age two to five years The sample size calculation was based on estimated prevalence of IDA of 13% 22 and 69% 23 among Tanzanian children with SCA and Nigerian children respectively with 90% power at the 5% level of difference between the two groups in a two-tailed test. The haemoglobin genotype of each study subject and control was determined using cellulose acetate paper for electrophoresis in alkaline medium.

### Inclusion criteria

- Age six months to five years
- Confirmed Hb SS by electrophoresis
- Subjects in steady state i.e. absence of any crisis in the preceding four weeks and absence of any symptoms or sign attributable to an acute illness.^24^

### Exclusion criteria

- Denial of consent
- Children on long-term transfusion therapy
- Children who had received a blood transfusion within three months prior to the study
- Children with a history of prematurity or low birth weight
- Children who had received iron supplementation within three months prior to recruitment.

The inclusion criteria for the controls were the same as for subjects except that the haemoglobin genotype was AA and they had no symptoms or signs attributable to acute illness in the preceding four weeks. Also, the exclusion criteria for the controls were the same as for subjects except that the haemoglobin genotype was AA.

Five millilitres of blood were drawn from a convenient peripheral vein and was transferred into plain tubes. The vacuum tubes were labelled and placed in a cool box containing ice-packs. The samples were protected from light at all times using sheets of black plastic. They were transported to the Research Laboratory of the Department of Paediatrics, LASUTH within 8 – 12 hours.

After centrifugation the serum was separated and stored at minus 20ºC until assay. The unsaturated or latent iron-binding capacity (UIBC) was measured by spectrophotometric techniques. Transferrin saturation (expressed as percentage of total iron binding capacity) was calculated using the formula: 100 X the serum iron concentration divided by total iron binding capacity.^25^ Serum iron was measured using Iron Ferrozine test kit (Biosystems, Spain) while the TIBC was measured by using Iron/Total Iron Binding Capacity reagent set (TECO Diagnostics, USA). Serum ferritin was measured by using human ferritin enzyme immunoassay test kit (Diagnostic Automation, USA).

The diagnosis of IDA was established based on the following criteria: haemoglobin <11.0 g/dl^14^ plus two or more of the following: MCV <70fl, 26, 27 transferrin saturation (Ts) <16% 26, 27 or serum ferritin (SF) <25ng/dL.^26^, ^27^ Cut-off points were based on laboratory standards and other studies.^5^

Social class was determined from occupation and educational attainment of parents using the scheme proposed by Oyedeji.^28^ The subjects were classified into one of five classes (I – V) in descending order of social privilege. The data was analyzed using the Statistical Package for Social Science (SPSS) version 17.0. Level of significance was set at p < 0.05.

## Results

A total of 194 children, 97 each with genotype SS and AA respectively were recruited. The demographic characteristics of the study subjects are given in table 1.

Overall, the age of the subjects ranged from seven months to sixty months with a mean of 30.6 (±15.97) months: 32.1 ± 16.12 months and 29.2 ± 15.77 months for SS subjects and AA controls respectively (M-W U = 4143.50, p = 0.151). The median ages were 25.0 months and 26.0 months in SS subjects and AA controls respectively. Ninety-six (49.5%) of the study subjects belonged to the upper socioeconomic strata (Socioeconomic indices I and II), while 34.5% and 16.0% belonged to the middle (Socioeconomic index III) and lower (Socioeconomic index IV and V) socioeconomic strata respectively.

### Haematological and Serum Iron Profile of Study Subjects

The comparisons of the mean haematological and serum iron profile values between HbSS subjects and HbAA controls are shown in table 2. The mean haemoglobin concentration was significantly higher in HbAA controls (p = 0.000). The mean MCV and serum ferritin were significantly higher among HbSS subjects. Mean transferrin saturation was higher among haemoglobin genotype AA controls than their SS counterparts but the observed difference was not significant (p = 0.697).

### Prevalence of Iron Deficiency Anaemia Among Study Subjects

In table 3, the prevalence of iron deficiency anaemia based on individual indices is shown. A combination of low Hb concentration and low MCV was found in 17.5% and 25.8% of sickle cell anaemia subjects and controls respectively. Using the model 1 diagnostic criteria for IDA (low Hb plus low MCV and low ferritin), no HbSS subject was identified as having IDA as against 12 HbAA controls. On the other hand, using the model 2 (low Hb plus low MCV and low transferrin saturation), three HbSS subjects and seven HbAA controls were identified as having IDA. The model 3 (low Hb plus low ferritin and low tansferrin) identified no HbSS subjects as having IDA while six HbAA controls were identified. Six HbAA controls with IDA who had low serum ferritin levels also had low transferrin saturation. The diagnosis of IDA in research is made when any of Model 1, Model 2 or Model 3 are satisfied.

Thus, the overall prevalence of IDA was significantly higher among AA controls than SS subjects (14.4% Vs 3.1%, χ^2^ = 7.113, p = 0.008). Further, of the 17 HbSS subjects who screened positive for anaemia using a combination of low haemoglobin and low MCV, 3 (17.6%) truly had IDA in contrast to 14 of 25 HbAA controls (56.0%) - χ^2^= 6.1 7, p = 0.013.

Model 4 which required the presence of all four diagnostic criteria (low Hb plus low MCV, low ferritin and low transferrin) yielded identical results with model 3. It is noteworthy that among HbSS subjects, two children with low serum ferritin and thirteen with low transferrin saturation did not meet the full criteria for IDA. The corresponding figures for HbAA controls were fifteen children with low ferritin and fifteen with low transferrin saturation.

The prevalence rates of IDA according to age-group and haemoglobin genotype are shown in table 4. All three HbSS subjects with IDA were in the older age bracket (> 2 years to 5 years) whereas, the majority of HbAA subjects with IDA, 13 of 14 subjects (92.9%), were in the younger age group (< 2 years). Thus, in the younger age group, the prevalence of IDA was significantly higher among HbAA controls than HbSS subjects (χ^2^ = 12.81, p = 0.0003 with Yates correction). In the older age group the odds of having IDA was three times higher among HbSS subjects (OR = 3.13, CI = 0.314 – 31.194) but the difference was not statistically significant (χ^2^ = 0.261, p = 0.610 with Yates correction).

Twenty-nine study subjects with sickle cell anaemia reported history of previous blood transfusions prior to commencement of the study. Of these 29, two had IDA. These two transfused subjects had recurrent blood transfusion.

## Discussion

The present study shows that a combination of low haemoglobin concentration and low MCV was a better screening tool for IDA in HbAA subjects than in those with HbSS. This conclusion is justified by the finding that less than twenty percent of SCA children with a combination of low haemoglobin concentration and low MCV had IDA. On the other hand, more than half of HbAA counterparts had IDA. This may be due to the fact that sickle cell anaemia sometimes may be associated with relative microcytosis in the absence of iron deficiency, which is assumed to be the consequence of deficient haemoglobin production as a result of a reduced amount of iron been delivered to the marrow erythroid precursors.^29^ This may also be direct effect of thalassemia traits among the study subjects. The fact that the current study made no attempt to detect/exclude thalassemia traits among the study subjects is in fact an important limitation of this study.

The overall prevalence of IDA among children with sickle cell anaemia in present study was 3.1%. This finding corroborates that of other workers^6^, ^7^ that IDA was uncommon among children with sickle cell anaemia. The reported prevalence value from current study was lower than the 5% reported by Isah et al^6^ among children with sickle cell anaemia in Northern Nigeria. The observed difference is probably an effect of diagnostic criteria. The northern Nigeria study employed the additional use of bone marrow aspirate examination. It has been reported that infants and young children have little or no iron stored in the marrow.^30^ Thus some subjects with low or no stainable marrow iron without low body iron stores could have been classified as IDA in the northern Nigerian study. In contrast to reported low prevalence value from this present study a higher prevalence value of 31% was reported among twenty-four Nigerian children with sickle cell anaemia aged less than ten years.^31^ The observed difference is possibly an effect of sample size, age range of study group and diagnostic criteria used. The small sample size of twenty-four Nigerian children with sickle cell anaemia aged less than ten years in the study by Okeahialam^31^ may have produced exaggerated prevalence rates. The diagnostic criteria used for identifying children with IDA by Okeahialam et al^31^ are ratio of free erythrocyte protoporphyrin (FEP) to haem and the packed cell volume. There is very little information available regarding the measurement or possible clinical application of FEP/haem ratio among Nigerian children.^31^ Thus the degree of comparability of results with the findings of the current study is not established.

In comparison with haemoglobin SS subjects, children with haemoglobin AA had significantly higher prevalence of IDA. This finding is in line with observations of other workers confirming rarity of IDA among subjects with sickle cell anaemia.^6^, ^7^ At the Lagos State University Teaching Hospital, routine prophylaxis of antimalarials (daily proguanil 100 – 200mg) in addition to folate (5mg) is being currently offered to children with sickle cell anaemia accessing its services. Some studies have indicated that malaria control alone effectively reduces the prevalence of child-hood anemia.^32^ Considering that routine antimalarials in addition to folates given to children with sickle cell disease is being practiced in our locale, the prevalence of IDA among children with sickle cell anaemia will be reduced.

The prevalence of IDA of 27.1% for the haemoglobin AA controls under the age of two years was almost twice as high as the 14.9% earlier reported in Lagos among children of comparable age.^33^ The higher prevalence in the present study may be attributable to the IDA defining criteria. While the current study defined low ferritin levels as values below 25ng/dl, the earlier Lagos study used a figure of 16ng/dl.^33^ Similarly, the present study defined low transferrin concentration as values below 16% as against 10% by Fajolu et al.^33^ It is not surprising therefore that a higher prevalence rate was observed in the current study. The explanation for the finding of higher rate of IDA in young children below two years of age was a result of rapid growth which occurs at this age group that result in increased iron requirements with intake of frequently inadequate dietary iron.

Contrary to what was observed among the haemoglobin AA controls, IDA was only observed in haemoglobin SS subjects older than two years of age. This finding may be anecdotal as the figures obtained were too small for meaningful statistical analysis. This further emphasizes the need for a larger, collaborative study in order to clarify the situation.

The diagnostic model containing ferritin concentration (Model 1) had a lower degree of success in identifying IDA than the transferrin saturation containing model (Model 2). None of the three HbSS subjects who met the IDA criteria using the transferrin containing model had sufficiently low ferritin to also satisfy the alternative criteria. The number of affected patients was however too small to make conclusive remarks. Perhaps, a larger pool of patients from a collaborative, multicentre study would provide more useful information. Serum ferritin are limited because of varying ranges of sensitivities and specificities, as they may be modified by conditions other than iron deficiency such as SCD.^2^

With respect to HbAA controls, the performance levels of both models (i.e. transferrin saturation model and serum ferritin model) were satisfactory with the serum ferritin model identifying a larger number of subjects with IDA. It is therefore attractive to recommend it as the preferred model for diagnosis of IDA in subjects with HbAA. However, the overall yield of IDA using either of the models was relatively small and a larger study such as recommended above should provide more conclusive information.

A comment on the place of blood transfusion in preventing IDA especially in HbSS subjects is desirable at this point. It was observed that two of the three haemoglobin SS subjects with iron deficiency anaemia had a history of blood transfusions. The number of transfused subjects was however too small to conclude that blood transfusion conferred no protection against IDA. Due cognisance is paid to the fact that other workers reported that none of the thirty-two children with sickle cell anaemia studied who had a history of blood transfusions have IDA.^2^

It is pertinent to address the issue of possible routine use of iron supplements in children with sickle cell anaemia. Iron supplementation is indicated when diet alone cannot restore deficient iron levels to normal within an acceptable timeframe. The goals of providing oral iron supplements are to supply sufficient iron to restore normal storage levels of iron and to replenish haemoglobin deficits. In practice, iron supplementation is not usually offered in sickle cell anaemia for fear of iron overload resulting from an added effect of multiple blood transfusions. Our opinion is that iron supplementation should still not be offered routinely to children with sickle cell anaemia since IDA is uncommon among this group of children as evidenced in current study where only 3.1% of the studied 97 unsupplemented haemoglobin SS subjects were found to have IDA.

Intestinal parasitic infections, especially helminths, represent a major public health problem that increase iron deficiency anaemia in developing countries.^34^ Hookworms contribute to anaemia because it induces iron deficiency by chronic intestinal blood loss.^35^ Blood loss is caused predominantly by parasite release of coagulases, causing ongoing blood loss in the stool, rather than actual blood consumption by the parasite.^35^ For example, *A. duodenale* is estimated to cause up to 0.25mL of blood loss per worm per day.^35^ A hookworm burden of 40 – 160 worms (depending on the iron status of the host) is associated with iron deficiency anemia.^35^ Young children are at greatest risk for hookworm-associated iron deficiency anemia due to low iron stores resulting from diets insufficient to meet demands.^35^ The current study did not set out to determine aetiology for IDA among the study subjects.

Overall, IDA is uncommon in pre-school aged subjects with SCA. IDA occurred exclusively in HbSS subjects older than two years but almost exclusively in younger controls. It is observed that low MCV is a weak screening test for IDA among HbSS patients. The transferrin saturation model was a more successful method to identify IDA among children with sickle cell anaemia but there is a need for a collaborative study to draw conclusions from a larger pool of patients.

## Figures and Tables

**Table 1:** Demographic characteristics of the study population

Characteristics	AA	SS	ALL
**Gender**			
Male	49 (50.5)	49 (50.5)	98 (50.5)
Female	48 (49.5)	48 (49.5)	96 (49.5)
Total	97 (100.0)	97 (100.0)	194 (100.0)
**Age group (years)**			
**Male**			
≤2	24 (49)	24 (49.0)	48 (49.0)
>2 – 5	25 (51.0)	25 (51.0)	50 (51.0)
Total	49 (100.0)	49 (100.0)	98 (100.0)
**Female**			
≤2	24 (50.0)	24 (50.0)	48 (50.0)
>2 – 5	24 (50.0)	24 (50.0)	48 (50.0)
Total	48 (100.0)	48 (100.0)	96 (100.0)
**Socioeconomic index**			
I	18 (18.6)	13 (13.4)	31 (16.0)
II	36 (37.1)	29 (29.9)	65 (33.5)
III	27 (27.8)	40 (41.2)	67 (34.5)
IV	15 (15.5)	14 (14.9)	29 (14.9)
V	1 (1.0)	1 (1.1)	2 (1.0)
Total	97 (100.0)	97 (100.0)	194 (100.0)

NB: Values in parenthesis are % of row total

**Table 2:** Haematological and serum iron profile of study subjects

	SS Mean (SD)	AA Mean (SD)	p-value
**Hb concentration (g/dl)**	6.74 (1.74)	9.67 (1.62)	0.000+
**MCV (fl)**	77.48 (6.10)	73.02 (5.73)	0.000+
**Serum ferritin (ng/dl)**	189.88 (104.28)	55.44 (54.20)	0.000*
**Transferrin saturation (%)**	26.00 (12.70)	28.83 (16.91)	0.697*

* = Mann-Whitney *U* test,

+ = Student t-test

**Table 3:** Distribution of individual criteria for diagnosing iron deficiency anaemia among study subjects according to haemoglobin genotype

Characteristics	SS (N = 97) No affected	%	AA (N = 97) No affected	%
**Hb <11g/dl**	92	94.9	82	84.5
**MCV <70fl**	17	17.5	25	25.8
**Hb <11g/dl and MCV <70fl**	17	17.5	25	25.8
**Serum ferritin <25ng/ml**	2	2.1	27	27.8
**Transferrin saturation <16%**	16	16.5	22	22.7
**Model 1**	0	0.0	12	12.4
**Model 2**	3	3.1	7	7.2
**Model 3**	0	0.0	6	6.2
**Model 1 or Model 2 or Model 3**	3	3.1	14	14.4
**Model 4**	0	0.0	6	6.2

NB: Model 1: Hb <11 g/dl, MCV < 70fl, and Serum ferritin < 25ng/ml. Model 2: Hb <11 g/dl, MCV < 70fl and Transferrin saturation < 16%. Model 3: Hb <11 g/dl, Serum ferritin < 25ng/ml and Transferrin saturation < 16%. Model 4: Hb <11 g/dl, MCV < 70fl, Serum ferritin < 25ng/ml and Transferrin saturation < 16%

**Table 4:** Prevalence of Iron Deficiency Anaemia among study subjects

Age group (years)	SS No in Group	No affected	AA No in group No affected	
≤2	48	0 (0.0)	48	13 (27.1)
>2 – 5	49	3 (6.1)	49	1 (2.1)

**Total**	97	3 (3.1)	97	14 (14.4)

NB: Values in parenthesis are % of number in group. Overall χ^2^ = 7.113; Overall p = 0.008; + = Chi-square test (χ^2^).
